# Optimized invertase expression and secretion cassette for improving *Yarrowia lipolytica* growth on sucrose for industrial applications

**DOI:** 10.1007/s10295-013-1323-1

**Published:** 2013-09-06

**Authors:** Zbigniew Lazar, Tristan Rossignol, Jonathan Verbeke, Anne-Marie Crutz-Le Coq, Jean-Marc Nicaud, Małgorzata Robak

**Affiliations:** 1 Department of Biotechnology and Food Microbiology, Wroclaw University of Environmental and Life Sciences, Chełmońskiego 37/41, 51-630 Wroclaw, Poland; 2 INRA, UMR1319 Micalis, 78352 Jouy-en-Josas, France; 3 AgroParisTech, UMR Micalis, 78352 Jouy-en-Josas, France; 4 CNRS, Micalis, 78350 Jouy-en-Josas, France; 5 Present Address: INRA, UMR1319 Micalis, AgroParisTech, 78352 Jouy-en-Josas, France

**Keywords:** Invertase, *Yarrowia lipolytica*, Sucrose, Citric acid, Secretion

## Abstract

*Yarrowia lipolytica* requires the expression of a heterologous invertase to grow on a sucrose-based substrate. This work reports the construction of an optimized invertase expression cassette composed of *Saccharomyces cerevisiae* Suc2p secretion signal sequence followed by the *SUC2* sequence and under the control of the strong *Y. lipolytica* pTEF promoter. This new construction allows a fast and optimal cleavage of sucrose into glucose and fructose and allows cells to reach the maximum growth rate. Contrary to pre-existing constructions, the expression of *SUC2* is not sensitive to medium composition in this context. The strain JMY2593, expressing this new cassette with an optimized secretion signal sequence and a strong promoter, produces 4,519 U/l of extracellular invertase in bioreactor experiments compared to 597 U/l in a strain expressing the former invertase construction. The expression of this cassette strongly improved production of invertase and is suitable for simultaneously high production level of citric acid from sucrose-based media.

## Introduction

*Yarrowia lipolytica* is an attractive tool for microbial bio-oil production [1, 3], for the production of compounds of interest like citric acid (CA) [9, 17, 31, 37], erythritol [29, 30, 33], polyunsaturated fatty acids for human health industry [38], bio-plastics [13], biodiesel [1, 18], and for protein production [5, 7, 11, 19, 24, 34]. One of the strengths of this non-conventional yeast is its use as a model for metabolic function studies [2, 24]. Therefore, its genome has been sequenced and numerous genetic tools have been developed [4, 5, 8, 20]. *Y. lipolytica* is able to grow on a various range of substrates, from glucose to lipids and alkanes [2]. This is of particular interest for bioconversion and valorization of low-cost raw materials and industrial waste like glycerol, industrial fats, or molasses. *Y. lipolytica* is directly able to use most of these carbon sources, except for molasses, which has to be hydrolyzed first due to the absence of invertase in this yeast. Invertase cleaves sucrose, the main carbon source in molasses, into glucose and fructose. This is a bottleneck to the use of such material for cost-effective bio-conversion. In order to use *Y. lipolytica’s* biotechnological potential for bioconversion directly on sucrose-based substrates, heterologous invertase activity has to be introduced. Most of the *Y. lipolytica* strains used up till now have been derived from strains containing an expression cassette integrated at the *URA3* locus, allowing production of the *Saccharomyces cerevisiae* invertase Suc2p and the subsequent utilization of sucrose [25]. However, this construction proves to have some limitations in terms of regulation of invertase expression and secretion efficiency. In fact, the *SUC2* gene is driven by the XPR2 promoter, in which the expression is dependent on pH and on the presence of peptone or yeast extract (YE) [9, 19, 25, 26, 32]. This construction also possesses a hybrid secretion signal composed mainly of the pre-secretion sequence of *Y. lipolytica* *XPR2* (corresponding to the 23 N-terminal amino acids from Xpr2p) followed by the coding part of *S. cerevisiae* Suc2p starting at amino acid eleven, which has for consequence to remove most of the Suc2p secretion signal sequence [6, 25]. The secretion of this chimera is only partial, with around 10 % being found in the supernatant [17, 25].

Cost-effective industrial fermentations benefit from a fast process and a high yield of one or even multiple products. Recently, the principle of simultaneous co-production of the invertase, one of the most used enzyme in industry [16], and citric acid has been described [17]. We reasoned that optimizing expression and secretion of invertase in *Y. lipolytica* could allow better growth on sucrose-based raw materials for production of compounds of interest and allows obtaining a high yield of the enzyme easier to purify for industrial purposes.

To this end, a new optimized invertase expression cassette using a strong promoter and the *S. cerevisiae* Suc2p secretion signal sequence was developed, in order to improve *Y. lipolytica* capacity to grow on sucrose-based raw materials and to boost the production and secretion of invertase and the production of compounds of interest. An increase in Suc2p secretion capacity has been recently observed in *Y. lipolytica* by replacing the upstream signal sequence of Suc2p [14]. This work reports a comparative study for invertase secretion and activity, growth kinetics, sugar utilization, and citric acid production at the bioreactor scale of *Y. lipolytica* strains expressing the new optimized invertase expression cassette compared to strains expressing the former one.

## Materials and methods

### Plasmid construction

Plasmid JMP1047 (JMP62 URA3ex pTEF) derives from JMP803 [13] with a replacement of the pPOX promoter by the pTEF promoter [23] using *Cla*I and *Bam*HI restriction sites. Plasmid JMP1462 was obtained by cloning the secreted form of the *S. cerevisiae* invertase *SUC2* gene into JMP1047. *SUC2* was PCR amplified from SC288C strain genomic DNA using primers SUC2up (CGCAGATCTCACAATGCTTTTGCAAGCTTTCCTTTTCC) and SUC2down (GGTGCCTAGGCTGCCTATTTTACTTCCCTTACTTGGAACT) containing, respectively, *Bgl*II and *Avr*II restriction sites. The corresponding *Bgl*II-*Avr*II PCR fragment was cloned into JMP1047 previously digested with *Bam*HI and *Avr*II allowing the expression of *SUC2* gene under the constitutive pTEF promoter. The construction was sequence verified.

**Fig. 1:**
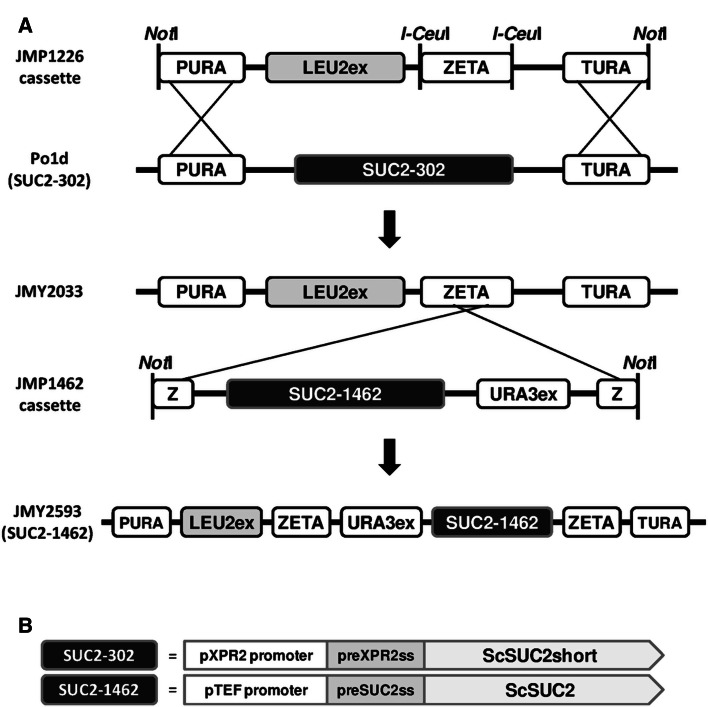
**a** Schematic representation of integration of *Not*I digested PTURA-LEU2ex-zeta cassette from JMP1226 by double crossing-over at the *URA3* locus of Po1d, giving rise to JMY2033, followed by single crossing-over of the SUC2-1462 cassette from JMP1462 at zeta platform giving rise to JMY2593. **b** Schematic representation of the 2 invertase expression cassette SUC2-302 and SUC2-1462. Signal sequence (ss); *S. cerevisiae* SUC2p (ScSUC2); truncated form of *S. cerevisiae* SUC2p deleted of the 11 first amino acids (ScSUC2short)

The zeta docking cassette for locus specific integration [5] was synthesized by Geneart (Life Technologies, Saint Aubin, France) and cloned at the *I*-*Ceu*I restriction site of a vector containing a 1-kb promoter and 1-kb terminator region of *URA3* surrounding the LEU2ex excisable marker and a *I*-*Ceu*I restriction site (unpublished data, Fig. 1). This construction gives rise to JMP1226 (PTURA3-LEU2ex-zeta, Table 1). This cassette allows the integration by double crossing-over at the URA3 locus of a LEU2ex-zeta docking platform (Fig. 1) providing a locus-specific integration site as previously described [5].

**Table 1:** Strains used in this study

Strains			
*Y. lipolytica*	Genotype	Invertase construction^a^	References
B56-5	*MATA, X*-*302:pXPR2::SUC2*	2X SUC2-302	[3]
PO1d	*ura3*-*302, leu2*-*270, xpr2*-*322*	SUC2-302	[1]
JMY2033	Po1d zeta platform	No invertase	This study
JMY2314	Po1d, *LEU2*-pTEF-RedsTar2	SUC2-302	[2]
JMY2529	JMY2314, *URA3*	SUC2-302	This study
JMY2531	JMY2314, *URA3*-pTEF-preSUC2-*SUC2*	SUC2-302, SUC2-1462	This study
JMY2593	JMY2033, *URA3*-pTEF-preSUC2-*SUC2*	SUC2-1462	This study

*E. coli*	Plasmid	Invertase construction^a^	Reference
JME1226	JMP1226 (PTURA3-LEU2ex-zeta)		This study
JME1047	JMP1047 (JMP62 URA3ex pTEF)	Empty vector	This study
JME1462	JMP1462 (JMP62 URA3ex-pTEF-preSUC2-*SUC2*)	SUC2-1462	This study

^a^Nomenclature used for invertase construction in results and discussion paragraph

#### *Y. lipolytica* strains used in this study

The *Y. lipolytica* strains used in this study are listed in Table 1. The strain B56-5 derived from A-101 [35], the other derived from the auxotrophic Po1d strain (Leu^−^, Ura^−^, derived from W29) described by Barth and Gaillardin [2]. JMY2529 and JMY2531 were respectively obtained by introducing the *Not*I digested and purified expression cassette from JMP62 URAex pTEF empty vector (JMP1047) and JMP62 URAex pTEF-preSUC2-SUC2 (JMP1462) into JMY2314, a LEU^+^ derivative of Po1d [15], by random integration using the lithium acetate transformation method described previously [10]. All of these strains are therefore prototrophe for leucine and uracile. In order to get a strain devoid of the former pXPR2-preXPR2-SUC2, the *Not*I digested and purified LEU2ex-zeta docking platform from JMP1226 was introduced at the *ura3*-*302* locus in Po1d by double crossing-over, deleting the former SUC2 expression cassette (see Fig. 1), giving rise to strain JMY2033. The strain has been verified for LEU^+^ and Suc^−^ phenotype and correct integration has been PCR verified. This strain now contains a specific zeta integration platform at the *ura3* locus (Fig. 1). JMY2593 was obtained by single crossing-over integration of the *Not*1 digested and purified expression cassette from JMP1462 (URA3ex pTEF-preSUC2-SUC2) at the *ura3::LEU2ex*-*zeta* locus of strain JMY2033 (Fig. 1). Correct integration was confirmed by PCR and SUC^+^ phenotype was verified. All the strains used in the following experiments are therefore prototrophic.

### Media and culture conditions

#### Growth in microtiter plates

Yeast strain growth in 96-well plates was performed in 200 μl of minimum medium YNB containing 0.17 % (w/v) yeast nitrogen base (without amino acids and ammonium sulfate), 0.5 % (w/v) NH_4_Cl, and 50 mM phosphate buffer (pH 6.8), with 0.5 % glucose or sucrose. Final concentrations of 0.1 and 0.01 % peptones or YE were added for pXPR2 promoter induction experiments; 24-h YPD precultures were washed and standardized to an 0D_600_ of 0.2. Triplicate experiments with each time 2–3 replicates for each strain/condition were performed at 28 °C under constant agitation with a Biotek Synergy MX microtiter plate reader (Biotek Instruments, Colmar, France), and was monitored by measuring optical density at 600_nm_ every 20 min for 40 h.

#### Bioreactor study

CA and invertase biosynthesis as batch cultures, were carried out during 72 h in 5-l stirred-tank reactors BIO-STAT B-PLUS (Sartorius, Frankfurt, Germany) with a 2-l working volume, at 30 °C, 800 rpm and aeration rate 0.36 vvm. Production media contained in 1 l of tap water: sucrose 100 g, NH_4_Cl 1.5 g, KH_2_PO_4_ 0.7 g, MgSO_4_ × 7H_2_O 1.0 g, YE 0.3 g, thiamine 3 × 10^−6^ g. Culture acidity was automatically controlled at pH 6.8 using 40 % (w/v) NaOH solution. Inocula consisted of 10 % of total working volume. The inoculum medium contained in 1 l of tap water: sucrose 50 g, NH_4_Cl 1.5 g, YE 1.0 g, peptone 1.0 g. The cultures were grown in 0.25-l flasks containing 0.05 l of medium on a rotary shaker (Elpan, Poznań, Poland), 240 rpm, 28ºC, 48 h.

For the analysis, 25-ml samples were taken and centrifuged 10 min at 5,000 rpm using 3–16 K centrifuge (Sigma, St. Louis, MO, USA). Supernatant and cells sediment were collected and used for further analysis. The first samples (time 0) were taken 10 min after culture inoculation. Regular samples were then performed over the 72-h bioreactor process as indicated in the figures. All experiments were performed in triplicate.

### Analytical methods

#### Biomass determination and intracellular invertase extraction

For dry biomass determination, the cells sediment of 3 × 5-ml culture sample was washed twice with distilled water, filtered on a 0.45-μm pore-size membrane, and dried at 105 ºC to a constant weight using the weight-dryer WPS 110S (Radwag, Radom, Poland). For kinetic analysis of intracellular invertase, 5 ml of collected samples was centrifuged 10 min at 5,000 rpm using 3–16 K centrifuge (Sigma, St. Louis, MO, USA), twice washed with 5 ml of distilled water and the enzyme was extracted by sonication (15 min, amplitude 100 %, 0.5-s intervals) using a SONOPLUS HD 2070 ultrasonic homogenizer (Bandelin GmbH & Co. KG, Berlin, Germany) followed by centrifugation. Supernatants were analyzed for invertase activity by measuring reducing sugars (fructose and glucose) released from sucrose (0.1 M) at pH 5.0 and 37 °C during 10 min.

#### Measurement of sugars and acids

CA, glucose, fructose, and sucrose were determined by HPLC (UltiMate 3000, Dionex-Thermo Fisher Scientific, UK) using an Aminex HPX87H column coupled to UV (210 nm) and RI detectors as described previously [17]. Isocitric acid was analyzed using the enzymatic method described by Goldberg and Ellis [12].

### Invertase activity

Extra- and intracellular invertase activity was measured in post-culture media and the cell’s extracts (supernatants) as described in Lazar et al. [17]. Briefly, enzymatic reaction was started by the addition of 0.2 ml substrate (0.1 M sucrose in H_2_O) to the mixture containing 0.1 ml of enzyme (diluted when needed), 0.1 ml of 0.1 M acetate buffer (pH 5.0) and 0.1 ml H_2_O maintained at 37 °C. Incubation was being continued for 10 min and then enzyme reaction was stopped by the addition of 1.5 ml of DNS reagent [21] and the sample was boiled (100 °C) for 5 min, cooled to room temperature, and filled with H_2_O to the final volume of 10 ml. Sample absorbance was measured at *λ* = 530 nm (Spectrophotometer, Marcel Media). One unit of activity (U) was defined as the amount of enzyme releasing 1 μmol of reducing sugars per minute in assay’s conditions.

## Results and discussion

Strain construction, validation of invertase activity and regulation of secretion.

The widely used version of *S. cerevisiae* *SUC2* invertase expression cassette in *Y. lipolytica* is under the control of the *Y. lipolytica* pXPR2 promoter [25], and its secretion signal sequence is a chimera of the pre-secretion signal sequence of *Y. lipolytica* *XPR2* in fusion with the *S. cerevisiae* *SUC2* sequence deleted of its own signal sequence (Fig. 1b). This construction allows only around 10 % secretion, while most of the enzyme remains associated with the cell [17, 25]. In order to increase the Suc2p secretion, the rate of sucrose cleavage, and subsequently the uptake of glucose and fructose, a new construction placing the full *S. cerevisiae* *SUC2* sequence including its own secretion signal sequence, under the strong and constitutive *Y. lipolytica* pTEF promoter was developed (see “Materials and methods” and Fig. 1b). For clarity and simplicity, the pXPR2-preXPR2-SUC2 construction will be named SUC2-302, while the pTEF-preSUC2-SUC2 construction will be named SUC2-1462 (Fig. 1b). The SUC2-1462 construction has been introduced in a strain expressing the former version SUC2-302 giving rise to the strain JMY2531, consequently expressing both forms of invertase expression cassette, as well as in a strain devoid of SUC2-302 giving rise to JMY2593, (see “Materials and methods” and Fig. 1a). As the former preXPR2-SUC2 hybrid secretion signal sequence from SUC2-302 is not efficient, the intermediate construction with the hybrid signal sequence upstream to pTEF was not developed.

**Fig. 2:**
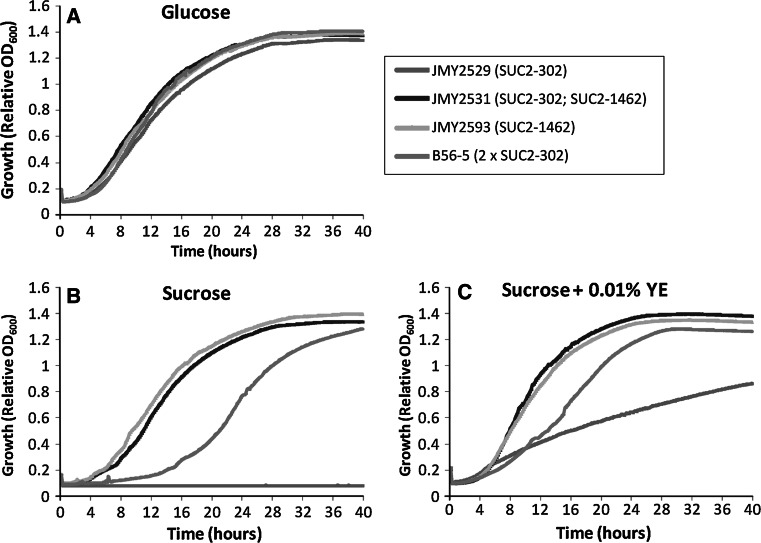
Growth curves on 96-well plates at 28 °C of SUC^+^ *Y. lipolytica* strains in minimum media with glucose (**a**) sucrose (**b**), or sucrose and 0.01 % YE (**c**). Representative growth curves corresponding to triplicate experiments with each time 2–3 replicates for each strain/condition are presented

These two strains were then compared with strain JMY2529 expressing one copy of SUC2-302 and strain B56-5 expressing 2 copies of SUC2–302 [17]. Growth capacity in minimum media with sucrose or glucose as the unique carbon source, supplemented with various concentrations of peptone or YE (known strong inducers of pXPR2) were evaluated in 96-well plates. Growths presented in Fig. 2 for each strain/condition are representative curves issue from multiple replicates (see “Materials and methods”). The invertase genotype of all these strains is summarized in Table 1. The four tested strains have a similar growth rate in glucose-based medium (Fig. 2a). The strain expressing only SUC2-302 in one copy (JMY2529) did not grow on sucrose-based medium in the absence of YE, while the strain expressing two copies (B56-5) grew with a strong delay compared to the two strains expressing SUC2-1462 (JMY2531 and JMY2593), which grew at a similar rate on glucose (Fig. 2b). In the same sucrose-based medium complemented with 0.01 % YE, the strains expressing SUC2-302 are growing accordingly to the number of invertase gene copies; JMY2529 expressing one copy is growing slowly, while the strain B56-5 expressing two copies has a higher growth rate, but still delayed compared to strains expressing SUC2-1462 (Fig. 2c). These data reflect the regulated pXPR2 or constitutively pTEF-driven expression. Similar results were obtained with 0.1 % YE or 0.1 and 0.01 % peptone (data not shown). Residual peptone or YE concentration is in fact sufficient for pXPR2 induction [32]. In sucrose-based media, the two strains expressing SUC2-1462 have similar growth, indicating that the additional expression of SUC2-302 in strain JMY2531 compared to JMY2593 does not confer any growth advantage (Fig. 2b, c). This indicates that SUC2-1462 alone is sufficient for optimal invertase activity in this condition and allows reaching the maximum growth rate on sucrose. Considering that the fully induced pXPR2 promoter is at least as efficient as the pTEF promoter for enzyme production and secretion [23], we can considered that the higher growth the strain expressing one copy of SUC2-1462 compared to the one expressing one copy of SUC2-302 in the presence of YE or peptone in sucrose is indicative of a higher invertase secretion efficiency of the SUC2-1462 construction. This suggests that the *S. cerevisiae* Suc2p secretion signal sequence is more efficient than the *Y. lipolytica* Xpr2p pre-secretion signal sequence for *S. cerevisiae* Suc2p secretion. In that particular case, using a heterologous secretion sequence signal appears more efficient than the construction of a chimeric protein bearing a *Y. lipolytica* secretion sequence signal.

Extracellular and intracellular invertase activity, sugars consumption, and CA production at the bioreactor scale.

In order to confirm these differences in invertase secretion effectiveness, intra and extracellular invertase activity profiles as well as sugar consumption by the above strains were investigated at the bioreactor scale. Additionally, the capacity for simultaneous CA co-production was analyzed as a demonstration of the potential of these strains for production of compounds of interest using sucrose as a carbon source.

**Fig. 3:**
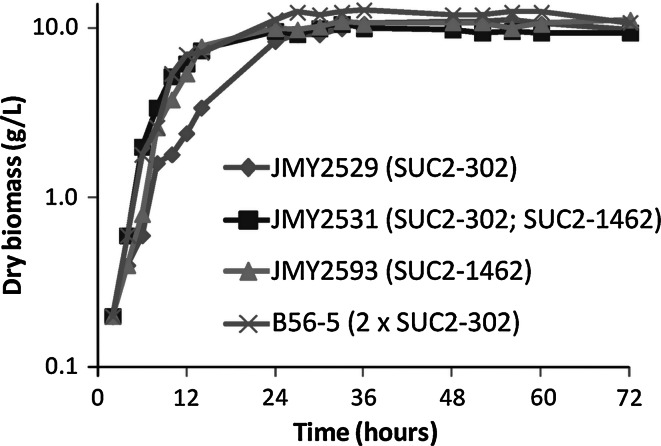
Growth kinetics of SUC^+^ *Y. lipolytica* strains in medium with sucrose as the carbon source in a 5-l bioreactor. Biomass accumulation during cultures is presented on log_10_ scale

To begin with, the growth kinetic of the four strains described above was determined by following biomass production in a 5-l stirred-tank bioreactor with sucrose as the sole carbon source and containing YE (0.03 % final concentration) as inducer of pXPR2 promoter for SUC2-302 cassette. In this experimental setup, strains JMY2531, JMY2593, and B56-5 have a similar growth rate and reach stationary phase within 14 h, whereas strain JMY2529, expressing only one copy of SUC2-302, has a slower growth rate and reaches stationary phase within 24 h but with a similar final cell density (Fig. 3). Strains JMY2531 and JMY2593 have maximum growth rates of 0.139 and 0.161, respectively. The strain B56-5 has a maximum growth rate of 0.132, while the strain JMY2529 has a growth rate of only 0.096 (Table 2). This is in line with what has been seen in microtiter plates, except that growth of JMY2529 is less delayed and that of B56-5 is not delayed compared to JMY2531 and JMY2593 in the bioreactor.

**Table 2:** Biomass, CA, ICA, extra- and intra-cellular invertase production parameters in a bioreactor with sucrose as the carbon source of the different *Y. lipolytica* SUC^+^ strains

Parameter	Biomass		Citric acid			Isocitric acid		Extracellular invertase		Intracellular invertase		Invertase	
	X	μ_max_	CA	Y_CA/S_	Q_CA_	ICA	ICA	EI	Q_I_	II	Q_I_	EI + II	EI
Strain	[g/l]	[1/h]	[g/l]	[g/g]	[g/l × h]	[g/l]	[%]	(U/l)	[U/l × h]	U/l	[U/l × h]	(U/l)	[%]
Y2529	9.8 ± 0.41	0.096	38.03 ± 0.84	0.50 ± 0.01	0.53 ± 0.01	0.75 ± 0.02	1.93	597 ± 53	8.29 ± 0.74	4,377 ± 621	60.8 ± 8.6	4,977	11.9
Y2531	9.4 ± 0.40	0.139	48.82 ± 1.22	0.58 ± 0.01	0.68 ± 0.02	0.41 ± 0.01	0.83	3,486 ± 428	48.42 ± 5.94	14,890 ± 1,334	206.8 ± 18.5	18,376	18.9
Y2593	11.2 ± 0.38	0.161	39.87 ± 0.75	0.50 ± 0.01	0.55 ± 0.01	1.06 ± 0.03	2.59	4,519 ± 445	62.76 ± 6.18	12,960 ± 1,841	180.0 ± 25.6	17,479	25.8
B56-5	10.8 ± 0.54	0.132	58.83 ± 0.88	0.65 ± 0.01	0.82 ± 0.01	0.75 ± 0.03	1.26	1,991 ± 262	27.65 ± 3.64	16,132 ± 2,135	224.1 ± 29.7	18,122	12.3

*CA* citric acid, *ICA* isocitric acid, *EI* extracellular invertase activity; *II* intracellular invertase activity, *Y*_*CA/S*_ citric acid yield from substrate, *Q*_*CA*_ citric acid volumetric productivity, *Q*_*I*_ invertase volumetric productivity, *X* biomass. Data presented are average of three replicates with standard deviation

### Invertase activity

**Fig. 4:**
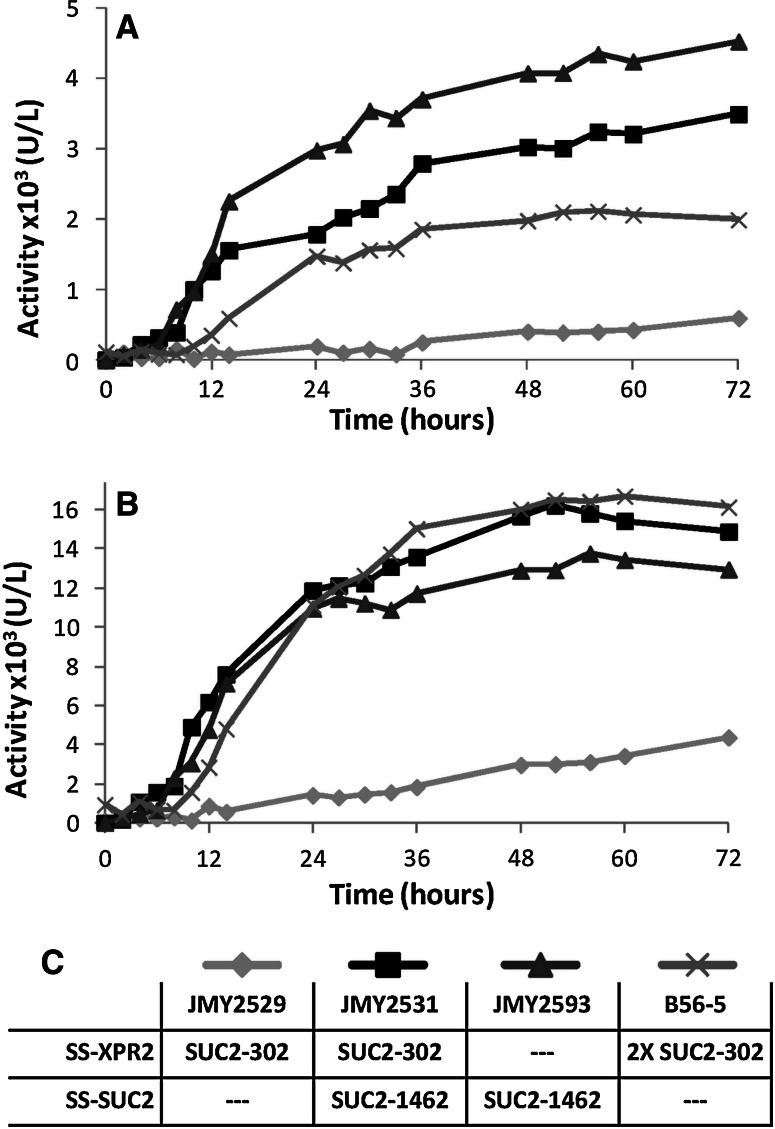
Kinetics of invertase activity in bioreactor of *Y. lipolytica* SUC^+^ strains in minimum medium with sucrose as the carbon source. **a** Extracellular invertase activity. **b** Intracellular invertase activity. Graphs presented are the average of three replicates. **c** Graphic legend and *SUC2* signal sequence (SS) present in each strain

Extracellular and intracellular invertase activities were evaluated in the bioreactor process. Extracellular invertase activity increases rapidly for strains JMY2531 and JMY2593 expressing SUC2-1462, whereas it is slower for strain B56-5 expressing two copies of SUC2-302 and stays very low for strain JMY2529 expressing one copy (Fig. 4a). This is in line with the higher secretion capacity of SUC2-1462 construction hypothesized previously. Similarly, the strain JMY2593 reaches the highest level of extracellular activity after the 72-h fermentation with 4,519 U/l, and strain JMY2529 the lowest with 597 U/l (Fig. 4a; Table 2). On the contrary, the increase in intracellular invertase activity is very similar between the strains B56-5, JMY2531, and JMY2593, and stays low for JMY2529 (Fig. 4b). Overall, JMY2593 ended with a slightly lower intracellular activity after the 72-h fermentation compared to B56-5 and JMY2531 (respectively 12,960, 16,130, and 14,890 U/l), while JMY2529 stays much lower with 4,380 U/l (Fig. 4b; Table 2). Considering the sum of intracellular and extracellular invertase activity (Table 2), JMY2531, JMY2593, and B56-5 have similar production levels. However, the proportion of secreted invertase for JMY2593 is much higher (25.8 %). It should be noted that the total activity of both *SUC2* constructions is not cumulative; the sum of total invertase activity of strain JMY2529 and strain JMY2593 does not correspond to the total invertase activity of JMY2531 combining both forms. Expressing simultaneously SUC2-302 and SUC2-1462 leads to a surprisingly lower extracellular invertase activity compared to JMY2593 expressing only SUC2-1462 (Table 2). It can be speculated that the presence of the secretion signal of SUC2-302, which has a defect in secretion capacity, may interfere with the secretion of the SUC2-1462 by overloading the secretion pathway. The other alternative is that the genetic environment of the integration site for SUC2-1462 cassette may impact the Suc2p expression/secretion level between these two strains. This also confirms that one integrated copy of SUC2-1462 alone is sufficient for optimal growth on sucrose (Fig. 2b, c). Similarly, strain B56-5, with two copies of SUC2-302, does not specifically produce two times more invertase compared to JMY2529 expressing only one copy (Table 2), a phenomenon that has previously been observed [17]. However, the overall proportion of secreted invertase is similar (11.9 and 12.3 %, respectively). By comparing JMY2529 and JMY2593 (which are expressing each only one form of invertase and which have the same genetic background) the last strain (expressing SUC2-1462) produces and secretes more invertase in terms of units per liter. Intracellular enzyme activity is 3.5 times higher and extracellular 7.5 times higher, which represents a massive improvement of invertase production. It can be concluded from this data that the secretion signal of SUC2-1462 is much more efficient than for SUC2-302. Hong and collaborators [14] very recently determined intracellular and extracellular invertase activity as a consequence of different variants of sequence signal in *Y. lipolytica*: the xpr2p prepro sequence signal followed by mature Suc2p, and native Suc2p with its own sequence signal, both under the hybrid strong promoter FBA1in. They end up with similar conclusions on secretion efficacy of the native Suc2p secretion signal sequence. However, they detected only the invertase activity extracellularly, which has been around 100 times lower than in our case in terms of U/l. This probably results from sampling time point differences and growth conditions, as they were measuring invertase activity in exponential growth phase at OD around 1.4–1.8, in flask, while data in Table 2 correspond to 72-h growth in bioreactor, corresponding to approximately OD = 30. Presented kinetics of invertase activity (Fig. 4a, b) have reveal that at the beginning of the exponential phase (6–8 h), extracellular invertase activities are very low within values in units per liter, in the same range of what Hong and collaborators [14] observed at a similar growth stage. However, it appears that in the bioreactor process, extracellular invertase rapidly reaches a much higher level (Fig. 4a). On the other hand, intracellular activities are already 3–5 times higher than in Hong et al.’s [14] study, even in the exponential phase. The presence of the second ATG allowing production of the minor cytosolic form in *S. cerevisiae* lacking the secretion signal sequence may explain the presence of such an intracellular invertase activity. Hong and collaborators [14] had a similar construction and failed to detect such activity. However, growth parameters in the bioreactor process allow reaching a much higher rate of invertase production, which can overload the secretion system. A more probable hypothesis is that a large part of the over-secreted invertase stays locked in to the periplasmic space during external invertase recovering experiments (supernatant), and is therefore attributed to the cell’s extract fraction. Preliminary protoplastization experiments revealed a very high release of invertase activity, thus strengthening this hypothesis (data not shown). However, we cannot exclude that the genetic environment of the cassette integration site might affect the expression of *SUC2*. Moreover, it cannot be excluded that the different genetic background between the strain used by Hong et al. [14] and strains described in this study may also affect the invertase secretion profile.

### Sucrose hydrolysis and sugar utilization

**Fig. 5:**
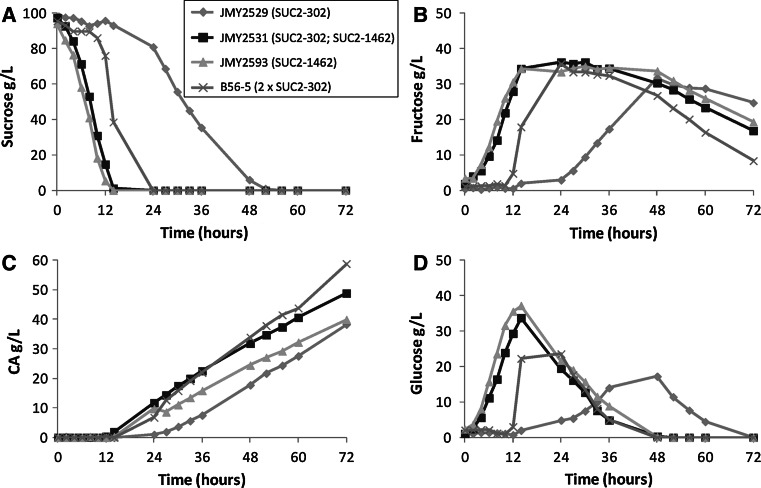
Sugars and CA concentrations during growth of *Y. lipolytica* SUC^+^ strains in a bioreactor with minimum medium and sucrose as the carbon source. **a** Sucrose hydrolysis kinetics. **b** Glucose concentration. **c** Fructose concentration. **d** CA concentration. Graph presented are average of three replicates

Invertase secretion allows sucrose hydrolysis and leads to glucose and fructose appearance in the medium, which are subsequently uptaken by the yeast. In the bioreactor study, the rate of sucrose hydrolysis and the subsequent glucose and fructose disappearance from the medium were also analyzed. Sucrose is hydrolyzed at different rates by the four invertase-expressing strains. Sucrose degradation rate (R_s_ in g/l/h) calculated for JMY2529 is the slowest one (*R*_*s*_ = 2.50) with sucrose being fully hydrolyzed after 52 h (Fig. 5a). It is faster for B56-5 (*R*_*s*_ = 6.15), where sucrose is fully hydrolyzed after 24 h (Fig. 5a), and even faster for JMY2531 (*R*_*s*_ = 7.63) and for JMY2593 (*R*_*s*_ = 7.07), which hydrolyses sucrose within 14 h (Fig. 5a). These last two strains have a very similar profile for sucrose hydrolysis. The profile of strain B56-5 is slightly different. It has a slow hydrolysis rate at the beginning of the growth until around 12 h when the hydrolysis rate increases. However, it does not affect growth compared to JMY2531 and JMY2593 (Fig. 3). These sucrose hydrolysis data are in line with the extracellular invertase activity level of these different strains. At mid-exponential phase, both strains JMY2531 and JMY2593 have a high and similar extracellular invertase activity and present a rapid sucrose cleavage, JMY2529 has very low activity and a low sucrose cleavage activity, with B56-5 being in between (Fig. 4a). JMY2531 and JMY2593 have the same sucrose hydrolysis rate, which confirms that expression of SUC2-1462 cassette alone is sufficient for maximum hydrolysis rate in that condition.

In parallel, glucose and fructose concentration in the medium were analyzed. The observed concentration of monosaccharides is a combination of the amount released from sucrose cleavage and that consumed by the yeasts (Fig. 5b, d). For all strains tested, it was observed that when sucrose is still present in the medium, glucose and fructose are consumed simultaneously and at the same rate, as glucose and fructose are present at equal concentrations in the medium. When sucrose is almost exhausted, glucose is rapidly consumed while fructose is not. In fact, fructose is only consumed when glucose is almost exhausted and at a much lower rate than glucose, independent of the strain tested (Fig. 5b, d). This later observation on glucose and fructose consumption has been previously mentioned [9, 17, 22]. It appears that fructose can only be used when glucose is at a very low concentration or if sucrose is present in the medium. This implies a complex regulation of glucose and fructose transporters’ activities. Identification of those potential transporters and their regulations will help in understanding this phenomenon.

#### Citric acid production

To validate the industrial potential of these strains for production of compounds of interest on sucrose-based substrates, the capacity of CA biosynthesis was also investigated. The Polish origin strain A-101 (B56-5 parental strain) and French origin strain W29 (JMY2529, JMY2531, and JMY2593 parental strain) have been historically selected for CA production [2, 36]. Strain B56-5 is a particularly high CA-producing strain already used for laboratory-scale production of this compound [17]. All of the four strains are able to produce large amount of CA in bioreactor condition with low isocitrate by product release (Table 2). The strain B56-5 stays the highest CA producer with 58.05 g/l after 72 h. Despite higher invertase secretion and sucrose degradation rates, strains JMY2531 and JMY2593 produce less CA, and JMY2529 is the lowest producer with a delayed production, probably due to a lower growth rate (Table 2; Fig. 5c). CA concentration at the end of the 72-h fermentation is inversely proportional to the remaining carbon source in the medium, which is only fructose at that time (correlation coefficient of −0.96). This is actually true from the beginning of the production of CA, considering the sum of sucrose, glucose, and fructose as the carbon sources (correlation coefficient of −0.94). The higher fructose utilization rate of B56-5 (Fig. 5b) might therefore explain its higher CA production compared to JMY2531-JMY2593. Thus, it could be speculated that after a long period, complete utilization of fructose for all strains might lead to similar citric acid production. However, the strain JMY2593 produces surprisingly less CA compared to JMY2531 at 72 h, while they share the same genetic background and a similar growth rate. JMY2593 ends up with a slightly higher biomass, which had partially redirected the carbon flux through biomass rather than CA production. However, it cannot be excluded that SUC-1462 integration locus differences between these two strains may have affected the CA production profile. Overall, CA yield from sucrose in these experiments (0.5–0.65 g/g; Table 2) are slightly lower than what has been obtained on sucrose with a CA overproducer mutant derivative of the German H222 strain by Förster et al. [9], but similar to what has been obtained on glucose with the Greek ACA-DC50109 wild-type strain [28], or the Polish A-101 wild-type strain [31]. It should be noted that at the end of the bioreactor experiments presented here, a significant quantity of fructose remains in the medium, and CA yield from sucrose is probably underestimated. Indeed, the yield could reach up to 0.85 g/g in some condition from the French wild-type strain W29 [27]. These data support the fact that sucrose is a good substrate for CA production by *Y. lipolytica* and that our new strains are suitable for such conversion.

## Conclusions

The new invertase expression cassette developed here allows a strong secretion of invertase in the medium and consequently a rapid cleavage of sucrose into glucose and fructose independent of any inducing condition and consequently not subject to inhibition by the medium composition. It opens the way for a rational utilization of *Y. lipolytica* in industrial fermentation using cheap sucrose-based substrates like molasses. It extends the panel of carbon sources to sucrose and molasses not only for CA production but also for lipid production or other compounds of interest that *Y. lipolytica* is able to produce. Moreover, the high level of invertase secretion will allow simultaneously setting up of invertase enzyme purification from post-culture medium for potential industrial applications. Co-production of CA has been achieved with all the strains tested. Although CA production starts earlier with the new expression invertase cassette, the final yield does not depend on the rate of sucrose hydrolysis. At this stage, it appears that the fructose utilization rate in stationary phase might be the limiting step for more efficient CA production. Expressing this new cassette in a A-101 background strain, which seems to have a better fructose utilization rate, would probably lead to an optimized strain for simultaneous high production of citric acid and invertase, with a faster and cost-effective bio-conversion process, particularly on sucrose-based substrates. Another approach will be to improve or deregulate fructose transport by genetic engineering of such potential transporters and regulators, which remain to be identified.
